# Case report: A rare immune-related adverse effect: hepatic cavernous hemangioma induced by camrelizumab

**DOI:** 10.3389/fimmu.2024.1387465

**Published:** 2024-04-05

**Authors:** Chuantao Zhang, Guanqun Wang, Ning Liu, Tianjun Li, Jingjuan Zhu, Helei Hou

**Affiliations:** ^1^ Department of Oncology, The Affiliated Hospital of Qingdao University, Qingdao, Shandong, China; ^2^ Department of Pathology, The Affiliated Hospital of Qingdao University, Qingdao, Shandong, China

**Keywords:** camrelizumab, adverse event, case report, visceral hemangioma, immunotherapy

## Abstract

**Background:**

Camrelizumab, a programmed death 1 (PD-1) inhibiting antibody, has demonstrated efficacy in various malignancies and received approval in multiple countries. Despite its therapeutic benefits, camrelizumab is associated with a unique spectrum of immune-related adverse effects (irAEs), predominantly reactive cutaneous capillary endothelial proliferation (RCCEP). However, visceral manifestations of such endothelial proliferations, particularly hepatic cavernous hemangiomas, have not been extensively documented.

**Methods:**

This case series retrospectively reviews six patients who developed hepatic hemangiomas following treatment with camrelizumab in combination with other chemotherapeutic agents. The series highlights the clinical course, imaging findings, management strategies, and outcomes associated with this complication. A detailed analysis was conducted to discern the potential causal relationship between camrelizumab therapy and the development of hepatic hemangiomas.

**Results:**

All six patients, after varying cycles of camrelizumab-based therapy, presented with hepatic lesions identified as cavernous hemangiomas on imaging. These findings were atypical for metastatic disease and were further complicated by significant clinical events, including massive intra-abdominal bleeding post-biopsy. Discontinuation of camrelizumab led to a reduction in the size of the hemangiomas in two cases, suggesting a potential link between the drug and the development of these vascular lesions. The incidence of RCCEP remained high, and the use of other agents such as bevacizumab did not mitigate the occurrence of hepatic hemangiomas, indicating a possible unique pathogenic mechanism associated with camrelizumab.

**Conclusion:**

Hepatic cavernous hemangioma may represent a rare but clinically significant irAE associated with camrelizumab therapy. This series underscores the importance of vigilant monitoring and a high index of suspicion for atypical hepatic lesions in patients undergoing treatment with PD-1 inhibitors. Further studies are warranted to elucidate the pathophysiology of this complication and to establish guidelines for the management and surveillance of patients receiving camrelizumab.

## Introduction

Camrelizumab, a programed death 1 (PD-1)-inhibiting antibody, has been approved by NMPA in treatment of advanced lung adenocarcinoma (1), hepatocellular carcinoma (2), esophageal squamous cell carcinoma (3), et al, in P.R. CHINA. Additionally, based on a multicenter phase III clinical trial conducted in 13 countries, the Office of Orphan Products Development (OOPD) of the FDA has awarded Orphan Drug Designation (ODD) to camrelizumab for HCC (4). It also has shown efficacy in gastric cancer (5), cervical cancer (6–10). The immune-related adverse effect (irAE) of reactive cutaneous capillary endothelial proliferation (RCCEP) occurs in 63.3% (11)-88.6% (12) patients induced by camrelizumab, which is much higher than other PD-1 antibodies. Previous study indicated that the RCCEP occurred mainly on the skin surface, oral cavity, and nasal cavity. Although there are case reported (13) on hepatic hemangioma, its documentation in the literature remains sparse. This case series presented 6 cases of patients who experienced hepatic hemangiomas after multiple cycles of therapy including camrelizumab.

## Case description

### Case 1

**Figure 1:**
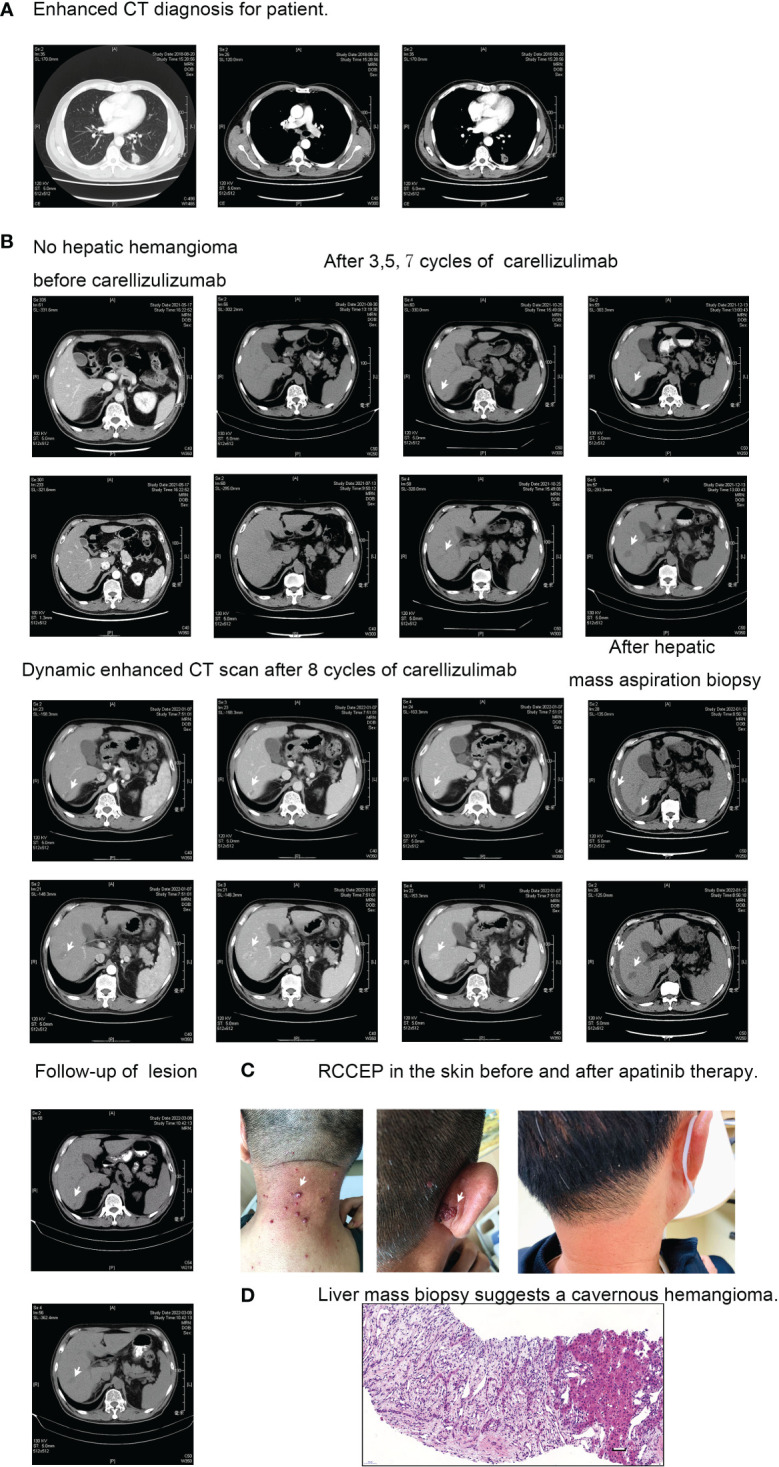
Diagnostic and treatment imaging. **(A)** Thoracic enhanced CT diagnosis. **(B)** Enhanced CT manifestation of hepatic cavernous hemangioma. **(C)** Skin reactions to RCCEP before and after apatinib treatment. **(D)** Pathological examination of hepatic cavernous hemangioma.

A 56-year-old male patient was diagnosed as primary lung cancer in July 2018. His clinical diagnosis was T1N3M0, stage IIIB adenocarcinoma with metastasis of hilar, mediastinal and supraclavicular lymph nodes (see **Figure 1A**). Meanwhile, the patient has a past medical history of hypertension and liver cysts.

The patient tested negative for EGFR mutation and ALK rearrangement, so they received a first-line therapy of pemetrexed, nedaplatin, and endostatin for four cycles from August 2018 to November 2018. During this time, they also underwent radical radiotherapy (56Gy/28f/5.5w) for the primary left lung lesion and lymph node metastases. They achieved a PFS of 7.0 months before a new metastatic lesion appeared in the right lung in April 2019. They were then given a second-line therapy regimen of pemetrexed, carboplatin, and bevacizumab for six cycles, followed by a maintenance therapy of four cycles of pemetrexed and bevacizumab. The best response was SD, and the PFS was 16.7 months. After the local lesion in the left lung progressed, the patient underwent palliative radiofrequency ablation. They were then given one cycle of the third-line regimen consisting of nab-paclitaxel and bevacizumab. Unfortunately, the patient developed a serious lung abscess and had to undergo anti-infective therapy for about two months. From May 19, 2021, to December 13, 2021, the therapeutic strategy was adjusted to nab-paclitaxel, bevacizumab (7.5mg/Kg), and camrelizumab (200mg, q3w), three weeks per cycle. The regimen was given for eight cycles, and the response was SD. As of March 13, 2022, no progression was observed.

**Figure 2:**
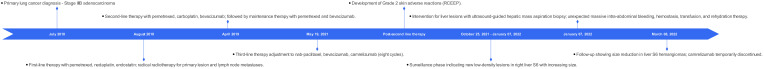
Treatment timeline for Case 1. Illustrates the sequence and duration of treatments administered to the first case.

The patient with advanced non-small cell lung cancer did not exhibit any skin or organ adverse reactions during the diagnosis until the failure of the second line of therapy. However, after one cycle of the third line therapy using nab-paclitaxel, bevacizumab, and camrelizumab, the patient developed Grade 2 skin adverse reactions (RCEEP), as shown in **Figure 1C**. Subsequently, an ordinary CT scan after five cycles of third line therapy revealed the emergence of two new low-density lesions in the right liver S6. These lesions gradually increased in size during surveillance from October 25, 2021, to January 07, 2022, as depicted in **Figure 1B**. In order to eliminate the possibility of mixed response, further examination was taken to verify the pathology of that new lesions in right liver S6. An ultrasound-guided hepatic mass aspiration biopsy was carried out on January 07, 2022. An unexpected massive intra-abdominal bleeding was observed after biopsy. Accordingly, the patient received hemostasis, transfusion, and rehydration therapy. The dynamic enhanced CT scan also showed typical progressive contrast filling in features of cavernous hemangiomas (see **Figure 1B**). The finally pathological diagnosis of the biopsy specimens was verified to be cavernous hemangiomas (see **Figure 1D**). Afterward, the camrelizumab was discontinued for one cycle. In Mar 08, 2022, an ordinary CT scan reported that the lesion of hemangiomas of liver S6 became smaller than before (see **Figure 1B**). The timeline of treatments for this patient is summarized in **Figure 2**.

### Case 2

A 61-year-old male patient was diagnosed with primary lung adenocarcinoma on July 15, 2019. The clinical staging was determined as T1N3M1, stage IV, characterized by extensive metastasis involving hilar and mediastinal lymph nodes, as well as bone. The patient was in excellent physical condition and had no previous medical record of hepatic hemangioma.

Commencing on August 8, 2019, the patient embarked on a first-line treatment regimen comprising gefitinib and bevacizumab. This therapeutic choice was guided by the presence of an EGFR exon 21 L858R submutation and the absence of an ALK-EML4 fusion rearrangement mutation, as revealed by genetic profiling. The treatment elicited a PR, extending the PFS to 9.7 months until the emergence of new metastatic lesions in the left scapula and chest bone, observed on May 29, 2019.

Subsequent to the detection of mutations in plasma via ddPCR, the patient was transitioned to a second-line therapy, comprising osimertinib and bevacizumab. This regimen stabilized the disease, prolonging the PFS to 5.2 months, despite an increase in pulmonary metastatic lesions. Concurrently, the patient underwent palliative radiotherapy (50Gy/25f/5w) to alleviate pain associated with bone metastasis.

In the ensuing phase of his treatment journey, the patient was administered a third-line chemotherapy regimen consisting of pemetrexed, carboplatin, and bevacizumab, spanning six cycles. This treatment elicited a favorable response, culminating in a PR and a PFS duration of 4.75 months. Nevertheless, a notable disease progression was observed by April 12, 2021, characterized by an escalation in both the number and size of metastatic lung lesions.

Subsequently, the patient embarked on a fourth-line treatment protocol, receiving three cycles of nab-paclitaxel (260mg/m2) and bevacizumab (15mg/Kg) once every three weeks, from April 15, 2021, to June 02, 2021. Concurrently, a genetic analysis was performed on a second biopsy sample procured from a pulmonary lesion, revealing c-Met amplification. Additionally, the PD-L1 IHC test yielded a TPS of 80%, indicating a high level of PD-L1 expression.

The patient attained a PR upon completing two cycles of the therapy. However, post the third chemotherapy cycle, the patient encountered several adverse effects including leukopenia, thrombocytopenia, elevated aspartate and alanine aminotransferase levels, and fatigue. These side effects necessitated the cessation of nab-paclitaxel. Subsequently, the patient was put on a maintenance regimen of camrelizumab and bevacizumab, which spanned 16 cycles from June 25, 2021, to April 13, 2022. Following pulmonary progression, a fourth-line treatment comprising anlotinib and osimertinib was initiated and continued from May 30, 2022, to October 19, 2022, achieving the best response of SD. Unfortunately, the patient succumbed to advanced illness and COVID-19 complications on November 21, 2022.

Throughout the treatment journey from the diagnosis of advanced NSCLC to the failure of the third-line therapeutic regimen, no RCCEP was observed in the skin or any organ. Notably, after one cycle of the fourth-line therapy, which included nab-paclitaxel, bevacizumab, and camrelizumab, Grade 2 skin RCCEP manifested From August 2021 to May 30, 2022, apatinib (250mg qod) was administered to mitigate RCCEP-related bleeding. This treatment effectively reduced the size and intensity of the RCCEP on the skin. However, it did not significantly impact the lesions in the anus nor prevent hepatic hemangioma. After 12 cycles of the fourth-line therapy, which included camrelizumab, RCCEP emerged in the anus, oral cavity, and skin. The anal lesion progressively enlarged, reaching dimensions of 1.0cm×0.5cm, and was surgically excised on January 29, 2021. The postoperative pathology confirmed the presence of a capillary hemangioma.

**Figure 3:**
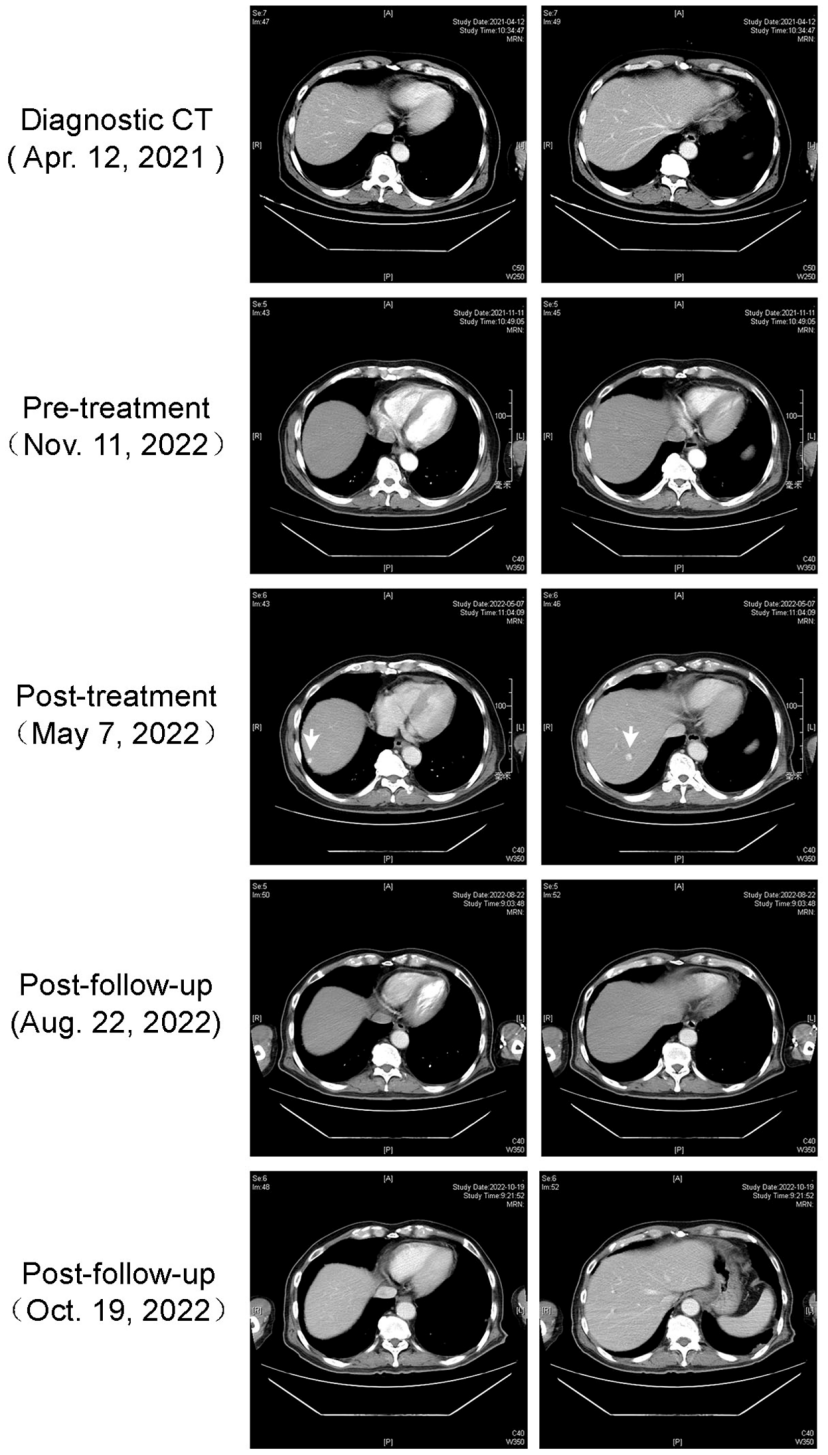
Case 2 hepatic cavernous hemangioma imaging. Enhanced CT visualization of hepatic cavernous hemangioma in the second case.

On May 7th, 2022, a dynamic enhanced CT scan disclosed the presence of three enhanced lesions. Specifically, one lesion was situated around the gastric fundus, while the remaining two were located in segment S7 of the right liver. These lesions were diagnosed as gastric fundus varicose veins and hepatic hemangiomas, respectively (as depicted in **Figure 3**). Notably, the patient did not exhibit cirrhosis, splenomegaly, ascites, or any other clinical indicators of portal hypertension. Remarkably, after a hiatus from camrelizumab treatment spanning 5.5 months, there was a discernible reduction in the size of the lesions. Throughout this interval, the patient showed no clinical signs of cirrhosis or portal hypertension.

### Case 3

A 65-year-old female presented with a two-month history of dry cough. She had a history of stage I hypertension, effectively managed with telmisartan, and no history of smoking. No history of hepatic hemangioma. The patient had lost approximately 3.5kg weight over a period of six months. On July 26, 2021, a chest CT scan revealed a mass in the left lower lobe of the lung, indicating lung cancer with mediastinal lymph node involvement and possible metastases. A biopsy on July 29 confirmed moderately to poorly differentiated adenocarcinoma. Genetic testing did not detect mutations in several common oncogenes (EGFR, ALK, ROS1, C-MET, NTRK, et al). The diagnosis indicates stage IV non-small cell lung cancer (adenocarcinoma), classified as T3NxM1. This includes metastases to the hilar lymph nodes, mediastinal lymph nodes, bones, and lungs, accompanied by carcinomatous lymphangitis in lung, pleural effusion, and pericardial effusion.

The first-line treatment began on August 3, 2021, with the PC regimen, which included pemetrexed and carboplatin. From August 25 to December 9, 2021, cycles 2-6 included camrelizumab and Bevacizumab combined with PC, resulting in SD. Maintenance therapy began on January 6, 2022, and continued until September 28, 2022, consisting of pemetrexed, camrelizumab, and bevacizumab. The treatment regimen was then adjusted starting from October 19, 2022, to August 2, 2023, with camrelizumab and bevacizumab. On October 21, 2023, a CT test showed PD again, and the second-line treatment began with a regimen including nab-paclitaxel plus bevacizumab for 4 cycles. The patient’s survival was last followed up in January 20, 2024.

**Figure 4:**
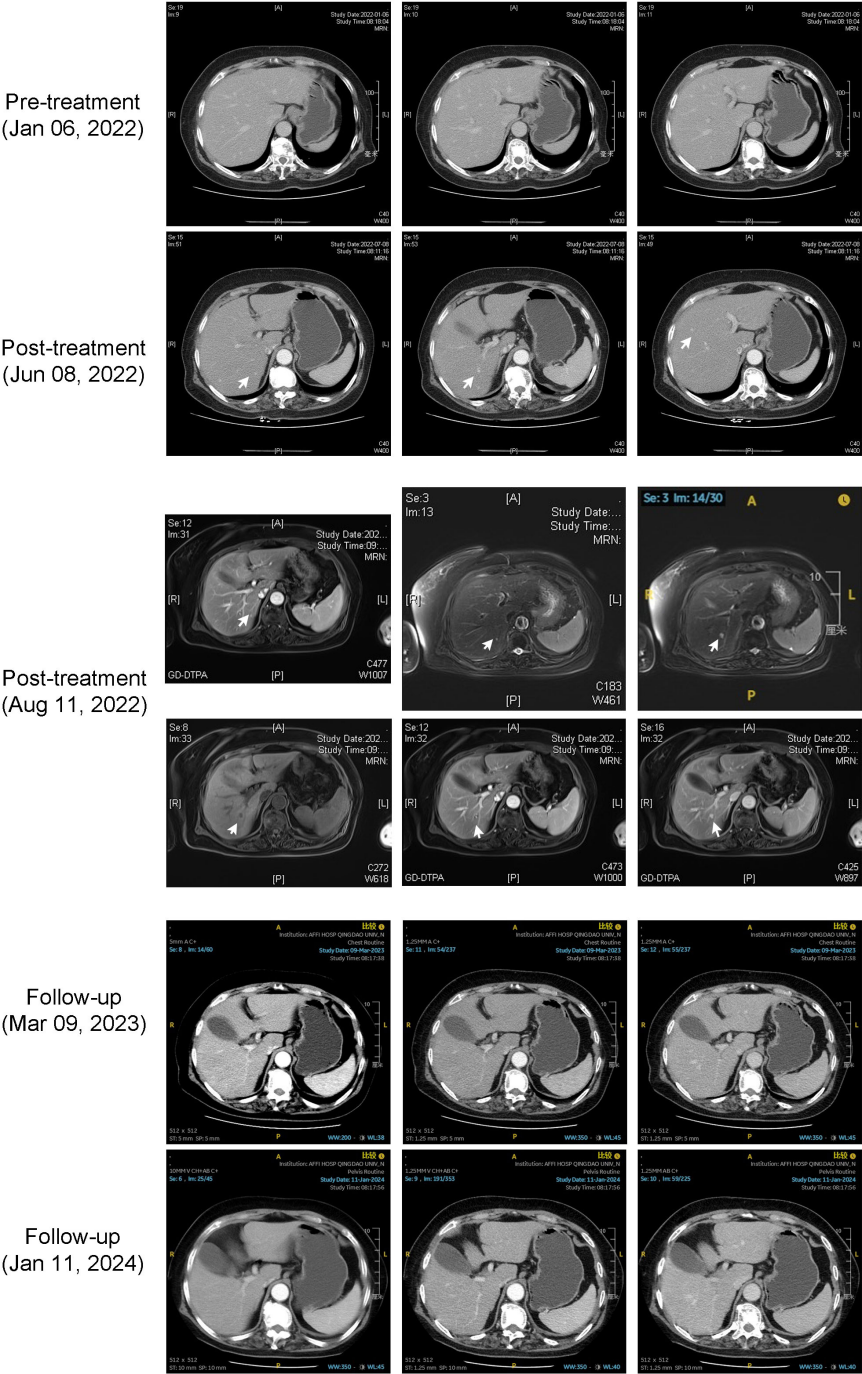
Case 3 hepatic cavernous hemangioma imaging. Combined enhanced CT and MRI manifestations of hepatic cavernous hemangioma in the third case.

During the treatment, an upper abdominal CT scan was conducted on July 8, 2022, which revealed the presence of several nodules in the right liver that exhibited dynamic enhancement. These nodules demonstrated a progressive enhancement pattern, indicating the presence of hepatic hemangiomas. This was confirmed by an MRI enhanced test conducted on August 11, 2022. Subsequent CT scans showed that the hepatic lesions had disappeared as of January 16, 2024 (see **Figure 4**). During the treatment sessions, no RCCEP was observed in the progression.

### Case 4

On January 21, 2021, a 56-year-old Chinese woman was diagnosed with gastric adenocarcinoma in the gastric body and admitted to a local hospital. A palliative resection was carried out on February 23, 2021, revealing a poorly differentiated adenocarcinoma upon postoperative pathology examination. Subsequent follow-ups detected metastasis in the supraclavicular and retroperitoneal lymph nodes, around the gastric fundus and cardia, and in the liver, classifying the patient’s condition as clinical stage IV. There was no documented history of hepatic hemangioma. Immunohistochemical (IHC) staining results were positive for HER2 (2+) and PD-L1 (CPS 5), but negative for MSH1, MLH, PMS, MSH2, MSH6, and AFP. A subsequent SISH test confirmed the positive HER2 status. From April 9, 2021, to June 26, 2021, the patient underwent four cycles of induction chemotherapy with tegafur, gimeracil, and oteracil potassium capsules, in combination with an oxaliplatin regimen (SOX). Trastuzumab and camrelizumab were added to the regimen starting from the second cycle. Maintenance treatment from July 7, 2021, to February 15, 2022, included a triplet regimen of tegafur, gimeracil, oteracil potassium capsules, trastuzumab, and camrelizumab, leading to tumor shrinkage and stabilization of the disease (SD).

After 12 therapy cycles including camrelizumab, dynamic contrast-enhanced CT scans of the abdomen on January 24, 2022, and February 21, 2022, revealed an abnormal intensification in the liver’s S7 segment from arterial to venous phase, suggestive of hepatic cavernous hemangioma (illustrated in the **Supplementary Figure 1**). This diagnosis was made despite the absence of typical dynamic manifestations such as peripheral globular enhancement and a centripetal fill-in pattern, with the attenuation of enhancing areas matching that of the aorta and blood pool. Unfortunately, follow-up data was unavailable after February 15, 2022.

### Case 5

A 54-year-old female presented with a year-long history of irregular vaginal bleeding, light red in color, with an unpleasant odor, accompanied by mild abdominal pain and difficulty in defecation. An initial consultation on May 7, 2019, led to a biopsy that confirmed poorly differentiated adenocarcinoma of the cervix. PET/CT scans showed extensive disease involvement, including the uterus, vagina, lungs, and lymph nodes. The diagnosis is confirmed as stage IVb cervical cancer, classified as adenocarcinoma, according to the FIGO 2009 staging system. There was currently no recorded history of hepatic hemangioma.

The patient received first-line treatment from May 22 to July 31, 2019. The treatment involved the TP regimen with endostatin (docetaxel, carboplatin, and endostatin). After two cycles, the patient showed PR. However, after four cycles, the patient’s condition worsened, showing PD. The patient then received second-line treatment from August 27 to December 20, 2019, which included nab-paclitaxel and cisplatin. After two and three cycles, the patient showed PR, and after four cycles, the patient’s condition became SD.

Maintenance therapy, comprising nab-paclitaxel and camrelizumab, was administered over four cycles spanning from January to April 2020. and then give maintenance therapy of camrelizumab. During this time, the patient showed stable SD. However, disease progression was observed in October 2020.

An enhanced CT scan in January 2021 revealed the presence of a hepatic hemangioma in the right hepatic lobe (Segment S6) (see the **Supplementary Figure 2**). Further chemotherapy commenced on January 28, 2021.

### Case 6

A 58-year-old male patient came in with a history of difficulty swallowing, hoarseness and a 3kg weight loss that had been going on for five months. The patient had a history of atrial fibrillation, valvular heart disease, smoking, and moderate alcohol consumption. An endoscopy revealed a cauliflower-like tumor in the esophagus which caused stenosis. The biopsy report confirmed that the patient had high to moderately differentiated squamous cell carcinoma. A PET/CT scan showed that the disease was extensive with esophageal, pulmonary, lymph node, and possible bone involvement. The diagnosis is definitively established as “esophageal squamous cell carcinoma, situated in the mid-thoracic region, classified as cTxNxM1, stage IV, accompanied by metastases to both lungs and bones.” There was no known record of hepatic hemangioma in the medical history. Between November 26, 2021, and March 6, 2022, the patient was treated with the TP regimen, which included nimotuzumab, resulting in stable disease after the 2nd and 4th cycles. From April 2 to April 26, 2022, the patient received treatment with nab-paclitaxel, camrelizumab, and nimotuzumab. Starting from May 16th, 2022, the patient underwent palliative radiotherapy for esophageal cancer. The total radiation dosage administered was 5600 cGy, which was delivered in 28 separate fractions.

Following four cycles of camrelizumab therapy, a dynamic contrast-enhanced abdominal CT scan conducted on July 10, 2022, revealed a low-density lesion in the anterior segment of the right liver lobe (Segment S8). During the venous phase, patchy low-density shadows were observed in the superior segment of the right anterior lobe, with the density in the delayed phase being slightly higher than that of the adjacent liver tissue, suggestive of an atypical hemangioma (see the **Supplementary Figure 3**).

## Diagnostic assessment

Case 1-6: Patients, ranging in age from 54 to 65 years and diagnosed with various advanced cancers (lung cancer, gastric adenocarcinoma, cervical cancer, and esophageal squamous cell carcinoma), underwent diagnostic evaluations confirming their primary cancers. All patients were later found to have developed hepatic cavernous hemangiomas following treatment with camrelizumab combined with other chemotherapeutic agents. Diagnostic methods included CT scans and MRI, revealing atypical hepatic lesions identified as cavernous hemangiomas, distinct from metastatic disease.

The characteristics of the six patients with hepatic cavernous hemangioma after camrelizumab therapy were summarized in **Table 1**.

**Table 1:** Characteristics of patients developing hepatic cavernous hemangioma following camrelizumab treatment.

Case No.	Age (years)	Gender	Diagnosis	Past interventions and their outcomes	Drugs of combined with camrelizumab	Line of camrelizumab	Camrelizumab cycles before hemangioma	Number of hepatic hemangiomas	Improvement or disappearance after discontinuation	RCCEP manifestations beyond visceral organs (Grades, Location)
1	56	Male	Lung cancer	1^st^ line Pemetrexed+Carboplatin+Bevacizumab(SD) 2^nd^ line Pemetrexed+ Carboplatin+Bevacizumab (SD)	Nab-paclitaxel + Bevacizumab (SD)	3	5	2	Yes	Yes (G2, Skin)
2	61	Male	Lung cancer	1^st^ line Gefitinib+Bevacizumab (PR) 2^nd^ line Osimertinib+Bevacizumab(SD) 3^rd^ line Pemetrexed+Carboplatin+Bevacizumab (PR)	Nab-paclitaxel, Bevacizumab (PR)	4	12	3	Yes	Yes (G2, Skin, Anus)
3	65	Female	Lung cancer	1^st^ line Pemetrexed+Carboplatin (SD)	Nab-paclitaxel+Bevacizumab (PD)	2	6	2	Yes	No
4	56	Female	Gastric cancer	No	Tegafur, Gimeracil, Oteracil, Trastuzumab, Oxaliplatin (SD)	2	12	1	UK	No
5	54	Female	Cervical cancer	1^st^ line Docetaxel, Carboplatin, Endostatin (PR)	Nab-paclitaxel+Cisplatin (SD)	2	4	1	UK	Unknown
6	58	Male	Esophageal cancer	No	Nab-paclitaxel+ Nimotuzumab (SD)	2	4	1	UK	Unknown

These patients, recognizing the importance of sharing their unique experience with hepatic hemangioma, contributed to our understanding without exhibiting symptoms. These cases underlines the necessity of vigilant monitoring for AEs like hepatic hemangioma, crucial for differentiating tumor progression and other conditions, thereby enhancing the collective knowledge base.

## Discussion

Prior to this study (13), a solitary article identified just two instances of hepatic hemangioma related to camrelizumab treatment, suggesting its rarity. The specific clinical features of such adverse effects remain largely undefined, due to a lack of comprehensive documentation on immune-related adverse effects (irAEs) manifested as reactive capillary endothelial proliferation or hemangiomas in internal organs caused by camrelizumab.

In this study, we present the first account of six patients who developed hepatic hemangiomas after undergoing 5, 16, 12, 14, and 4 cycles of camrelizumab therapy, respectively. In Yonglong Yu’s study (13), the number of therapy cycles reported shows similarities to those in our investigation.

In the cases of the 1st, 2nd, and 3rd patients, the occurrence of visceral hemangioma was noted post-treatment with a regimen including camrelizumab, bevacizumab, and chemotherapy. However, the diminution of liver lesions upon the cessation of camrelizumab leads us to posit that camrelizumab is the primary etiological factor for hemangioma formation.

The 4th patient exhibited hepatic hemangiomas subsequent to treatment with camrelizumab, trastuzumab, and chemotherapy. Regrettably, in cases 4, 5, and 6, the absence of follow-up imaging post-initial confirmation of hepatic hemangioma precludes definitive conclusions regarding lesion improvement post-camrelizumab discontinuation, thus suggesting a possible, albeit unconfirmed, correlation between camrelizumab and hemangioma emergence.

The discontinuation of camrelizumab in the 1st, 2nd, and 4th cases leaves open the question of potential hemangioma enlargement upon rechallenge with camrelizumab therapy. Moreover, literature lacks reports of RCCEP or hemangioma induction by bevacizumab (14), trastuzumab (15, 16), nimotuzumab (17), and chemotherapy, further complicating the assessment of these agents’ roles in such pathologies. In the study conducted by Yonglong (13), it was observed that the hepatic cavernous hemangioma resolved upon the cessation of camrelizumab treatment or when patients were switched to alternative PD-1 inhibitors. This outcome suggests a direct correlation between the medication and its adverse effects.

RCCEP occurrences outside visceral organs may not be associated with hepatic hemangiomas. The first and second cases reported RCCEP in the skin and anus, among other locations, which were not mentioned in the third case. Furthermore, the resolution of hepatic cavernous hemangiomas in the first and third cases after discontinuing camrelizumab underscores the potential reversibility of these effects, echoing findings from previous research (13, 18).

In exploring the pathogenic mechanisms of RCCEP, it is highlighted that camrelizumab’s binding to VEGFR2 may play a crucial role. Previous studies (5) suggest that RCCEP induced by camrelizumab may be mitigated by the VEGFR2 selective inhibitor, apatinib. However, the administration of bevacizumab in the 1st, 2nd, and 3rd patients did not curtail RCCEP occurrence, owing to bevacizumab’s neutralization of VEGF-A and its inability to block VEGFR2 activations instigated by camrelizumab.

Camrelizumab’s unique, low-affinity interaction with VEGFR2’s extracellular domain, functioning as an agonist, is pinpointed as a potential mechanism for capillary hemangioma induction (13, 19). This interaction stimulates vascular endothelial cells, fostering hemangioma formation (13). Bevacizumab (14), despite targeting VEGF-A isoforms, fails to inhibit VEGFR2 activation by camrelizumab, thus not preventing RCCEP or visceral hemangioma nor fully blocking camrelizumab-induced VEGFR2 activations.

VEGFR2 selective inhibitors like apatinib (18, 20) are documented to effectively reduce camrelizumab-induced RCCEP. Apatinib impedes VEGF-driven endothelial cell migration and proliferation, curtailing abnormal vascular growth typical of hemangiomas. However, despite apatinib’s efficacy in diminishing skin RCCEP (5, 19) in the 2nd case, it did not prevent visceral hemangioma occurrence, underscoring the need for further research to optimize apatinib dosing. Anlotinib (21–23), an orally administered multi-RTK inhibitor, demonstrates high VEGFR2 inhibitory potency in preclinical trials, akin to apatinib. Anlotinib’s potential in treating RCCEP or visceral hemangiomas post-camrelizumab discontinuation remains an open inquiry.

Elevated estrogen and progesterone levels, often associated with pregnancy and oral contraceptive use, have been implicated in liver hemangioma development in certain studies (24, 25). However, the patients in the fourth and fifth female cases, who are over 55 years old, postmenopausal, and do not use contraceptives, do not align with this demographic profile.

Including the patient perspective, our report details the patient’s subjective experience with hepatic hemangioma, noting the lack of symptoms and how this condition contributes to the differential diagnosis in the context of ongoing cancer surveillance.

Our study acknowledges several limitations: The lack of follow-up data for cases 4, 5, and 6 limits our ability to determine if hepatic hemangiomas can resolve spontaneously post-discontinuation of treatment. Additionally, the potential for hepatic hemangiomas to reoccur upon re-administration of the medication remains unclear for cases 1, 2, 3, 4, 5, and 6. The small number of cases may not fully represent the broader patient population. Moreover, the concurrent use of other medications obscures the precise determination of camrelizumab’s sole effect on the formation of hepatic hemangiomas. The atypical enhanced CT presentation of the hepatic hemangioma in the third patient suggests that incorporating enhanced MRI could provide more definitive diagnostic imaging evidence.

In clinical practice, the rarity of these irAEs demands heightened vigilance. Visceral hemangiomas, especially hepatic, might be misinterpreted as hepatic metastasis or disease progression in routine CT scans. Dynamic enhancement CT or MRI is pivotal for accurate differentiation of these hemangiomas from metastatic lesions, emphasizing the importance of considering such heptatic hemangiomas during camrelizumab therapy.

## Conclusion

Early identification of new hepatic cavernous hemangioma is important for evaluating the efficacy and safety of camrelizumab.

## Data availability statement

The datasets presented in this article are not readily available because of ethical and privacy restrictions. Requests to access the datasets should be directed to the corresponding authors.

## Ethics statement

The studies involving humans were approved by the Affiliated Hospital of Qingdao University. The studies were conducted in accordance with the local legislation and institutional requirements. Written informed consent for participation in this study was provided by the participants’ legal guardians/next of kin. Written informed consent was obtained from the individual(s) for the publication of any potentially identifiable images or data included in this article.

## Author contributions

CZ: Writing – original draft. GW: Investigation, Writing – review & editing. NL: Writing – review & editing. TL: Data curation, Writing – review & editing. HH: Conceptualization, Funding acquisition, Writing – review & editing. JZ: Conceptualization, Writing – review & editing.

## Supplementary material

Supplementary Figure 1 Case 4 Hepatic Cavernous Hemangioma Imaging Enhanced CT visualization of hepatic cavernous hemangioma in the fourth case.

Supplementary Figure 2 Case 5 Hepatic Cavernous Hemangioma Imaging Enhanced CT visualization of hepatic cavernous hemangioma in the fifth case.

Supplementary Figure 3 Case 6 Hepatic Cavernous Hemangioma Imaging Enhanced CT visualization of hepatic cavernous hemangioma in the sixth case.
